# Dr. O'Halloran on the Yellow Fever of the South and East Coasts of Spain

**Published:** 1823-09-01

**Authors:** 


***Remarks on the Yellow Fever of the South and East Coasts of Spain; comprehending Observations made on the Spot, by actual Survey of Localities, and rigorous Examination of Fact, at original Sources of Information.*** By Thomas O'Halloran, M. D. Member of the Medical Academies of Madrid and Barcelona. Octavo, pp. 208, with a large Plan of Barcelona; price 10s. 6d. boards. London, 1823.

The human mind, like the human body, is always most alive to the strongest excitant—and at the moment when we now write, the scourge of war has superseded in interest the ravages of Pestilence. How long will the beautiful vallies and populous cities of Mediterranean Spain continue to be desolated by the impious sword of man and the putrid breath of disease! Very long, we fear. While the human passions furnish incentives to the one, the physical elements around us will generate ample causes of the other.

The investigation of epidemic and endemic fevers has, for several years past, been carried on with such ardour and acrimony, that a kind of reaction or revulsion has now taken place in the public mind, and these inquiries are looked on with some degree of horror. It is not proper, however, especially in the Journalist, to give way to these temporary vacillations of taste or temper. What has been interesting once will be interesting again; and therefore it is his duty to place on record a faithful account of what passes in his day, for the benefit of posterity, if not for the gratification of the moment. But that taste must be fastidious, that mind contracted, that disposition selfish, which can turn a deaf ear or uninquisitive eye to the theatre, however far removed from his own vicinity, where fever annually sweeps thousands to the grave. The causes which first produce endemics and epidemics, and the laws by which they are afterwards propagated, cannot be too much or too often examined—especially as they are yet involved in great obscurity. This is the paramount reason of our entering into the subject as often as it presents itself—but there are other reasons, though minor, which stimulate us to this task on the present occasion. The two individuals, our countrymen, who have lately investigated the fever of Spain, were actuated by the most disinterested zeal, and undertook the dangerous and laborious office at their own personal risk, and at their own private expense —Government having granted little else than *permission* for the hazardous enterprize. To Drs. Jackson and O'Halloran, then, we affirm, the profession is under a debt, which ought to be repaid by at least an attentive perusal of their works. It is only by the zeal and intelligence of its individual members that the respectability of the profession itself can be sustained or promoted, and the examples of the two gentlemen in question deserve to be recorded in the most grateful manner, and held up as incitements to their brethren at large. Having made these few preliminary observations, we shall proceed to lay before our readers some account of Dr. O'Halloran's labours.

The work is dedicated, by permission, to His Royal Highness the Duke of York, and as this commander-in-chief has always been distinguished by his discrimination in rewarding merit, we hope His Royal Highness will not permit the present occasion to be regarded as an exception to the general rule of his conduct.

Dr. O'Halloran having spent some years in the West Indies, and there undergone, in his own person, a visitation of yellow fever—and having also served some time in North America, and at Gibraltar, he may be fairly allowed to be a proper person for the investigation in question. It will also be recollected by our readers that our author accompanied the venerable Dr. Jackson on a former occasion, and assisted in exploring the nature, causes, and treatment of the epidemic on another part of the Spanish coast.

The first chapter of the work contains a sketch of the medical topography of Barcelona and its environs, of which we deem it proper to take some notice. Catalonia, the province is bounded on two sides by the Pyrennees and Mediterranean. Its mountains are high and rugged, and intercepted by deep ravines, through which torrents descend impetuously in the rainy season. The elevated country, not mountain, is a light barren soil. The plains are numerous, small, and fertile. The country is watered by several rivers, of which the Ebro is the largest. The country contiguous to Rosas is alluvial, swampy, and extremely insalubrious, the inhabitants being severely harrassed by intractable, and often malignant, intermittents. To the eastward and northward of Barcelona there is no ostensible swamp, but water is found at all points there within three feet of the surface. The country to the *westward* of the city is watered by the Llobrigat River, and the principal part of the land contiguous to the river is swampy during the autumnal months, or rainy season, after which period it becomes partially dry, and admits of culture. The district adjacent to the mouth of the river, a mile in extent, "is actual swamp at all times."

"The noxious exhalations from this source not only produce disease among the peasantry, but the air of Monjui, a hill which overlooks Barcelona, is so deleterious in its qualities, as to render it necessary, on some occasions, to relieve the stationary guard every eight or ten days. The injurious impression of the exhalation arising from this swamp, manifests itself more conspicuously upon the summit than in the subjacent parts. Few escape disease who reside on the hill for any length of time; those who dwell on its skirts, or on the plain below, suffer little sickness comparatively, and experience little comparative mortality from their sickness; a fact proving, with a multitude of others, the superior force of the noxious influence of vapours, ascending from low, swampy grounds, to rocky and mountainous heights in their neighbourhood." P. 5.

This hill, which is 700 feet high, is not only useful in protecting the town and harbour from the enemy, but apparently in screening the town and environs from the unwholesome exhalations which arise from the swamp on the western side. Barcelona itself, situated on a bed of sand, nearly level with the sea, covers a space of two miles and a half in circumference. The houses are generally from three to five stories high—some of the apartments being large, spacious, and well ventilated—the windows of the sitting rooms descending to the level of the floor, and consequently the air circulating freely. The sleeping rooms, however, are too often small, dark, dirty, and without any means of ventilation. The houses have generally areas in the centre, but too small for light or ventilation. The under floor or flat is seldom inhabited, and abounds with exhalable moisture.

"The streets are extremely narrow, being seldom broader than two, three, or four yards, crooked, and intersected by a number of lanes and alleys. The sides of the streets are well paved: there are shallow drains in the centre for the conveyance of filth and water from the houses. They are covered with massive pieces of rock, three feet long, and about eight inches broad, so arranged as to leave interstices for the passage of water from the houses, during the prevalence of the autumnal and winter rains. The unwholesome vapours, produced by the filth which stagnates in the sewer, exhale through the interstices, so as to offend the sense of smell at all seasons of the year. They excite the disgust of the inhabitants who are accustomed to them; they not unfrequently turn the stomach of strangers.

"Upon the whole, when the narrowness of the streets, the excessive filth in the dwelling houses, the extraordinary construction of the sewers in the centre of the streets, and the dampness of the under stories are fully considered, there can be no surprise that the endemic disease should be of an aggravated kind, and, under the influence of a contingent epidemic, that it should be malignant." 8.

In Barcelona and environs ague is the most common form of disease—remittents are not rare—continued or gastric fevers are frequent at certain seasons—" and a form of malady called typhus by some, and at present known by the name of yellow fever, has appeared at intervals from the commencement of the present century." In 1803 and 1819 it prevailed to some extent—the mortality was considerable, but the disease was not then considered as arising from pestilential contagion.

In Barceloneta, a small, but regularly built town in the vicinity, the houses touch on the back part, and consequently are very deficient in ventilation. The apartments are small, and the whole interior miserable, on account of the filth of the inhabitants. It is also built on an alluvial soil, abounding at all seasons of the year with exhalable moisture. In this place, as may be readily supposed, the mortality was greater in proportion than in other and better situations. Yet the servants of the hospital did not suffer here; for of fifteen persons who were constantly employed in the care of the sick, only one contracted the malady.

" At the extremity of the harbour, between Barcelona and Barceloneta, the principal sewers of the city deposit their disgusting contents; and, as several of them unite at this point, they form a stream, the water of which is black as ink. It runs in an open channel for the distance of one hundred and fifty yards, before it falls into the sea. The effluvia which arise from this canal are disagreeable in the extreme, at all seasons of the year; they were unusually so before and during the epidemic, the drought being unusually great and of long continuance. Some of the families in the vicinity were so annoyed by it, as to be induced to quit their houses, and repair to the country. Besides the loathsome condition of the part alluded to, and the disagreeable effects it occasioned, not only to the inhabitants of the houses in the immediate neighbourhood, but to persons who accidentally passed that way, the sea for years past has gradually retired from the walls, having previously deposited sand in considerable quantity. The deposition is partially a swamp at the present time, from which, during the prevalence of hot weather, very offensive vapours arise." 12.

In the vicinity of this, the embouchure of the channel contained several condemned and half-rotten vessels, in a state of filthiness that nothing could surpass, and emitting a most offensive smell. From this account of the town harbour and locality, it will not appear surprising that fevers of a complicated and dangerous nature should arise occasionally there, without preternatural or foreign influence. In the summer of 1821, the first case was said to have appeared on shipboard, but this is not proved by good evidence. Wherever it began, it soon ravaged like a pestilence all the districts of this beautiful suburb, destroying life beyond any thing previously known in Spain.

The climate of Catalonia is very changeable, and the predominant easterly winds are always charged with moisture. In spring and summer the winds are hot and oppressive—the thermometer generally ranging between 77° and 86°—the mercury has even been known to rise as high as 90°. In fact, they have complete tropical temperature for some months of the year, and with it visitations of tropical diseases.

The second chapter of this work is entitled—"Importation of the Yellow Fever into Barcelona, in the Year 1821." The origin of yellow fever has occasioned much unprofitable discussion among the Spanish, as well as among our own physicians. Some of them consider it a disease of foreign origin imported through the medium of ships, clothes, or goods—others suppose it to be the endemic of the country, disfigured in its form through contingent causes, atmospheric or terrestrial.

The story of the Dolphin brig importing the yellow fever into Cadiz, in 1800, exhibits a remarkable illustration of the delusion which exists in Spain, and of the facility with which the credulous inhabitants of that country are imposed upon. This vessel sailed from the Havannah on the 26th of May, 1800; touched at Charlestown, in South Carolina, on the 30th, and there embarked three sailors, the town being in perfect health. She sailed again on the 11th of June, and arrived at Cadiz on the 6th of July. Twelve sailors composed the crew, and there were 22 passengers. Dr. Carrow, a physician, was among the latter. Three sailors died on the passage, in consequence of which the Board of Health at Cadiz interdicted all communication with the shore, the *captain* of the vessel stating that the men died of yellow fever. Dr. Carro, however, who attended these men, contradicted the assertion of the captain, and the whole of the passengers bore testimony in the same direction. Yet the opinion of the captain over-ruled that of the physician—consequently the importation of the disease was fixed on the Dolphin!

Each side of the question has its partizans in Spain; but the advocates for importation are most numerous, the voice of the people, and the policy of the Government, being all on that side. The others, who doubt this doctrine, dare not dispute the popular and political creed. "The delusion is an article of faith—is grateful to the people—is maintained by the Government for purposes—and those who have the pertinacity to oppose, or offer facts in refutation of opinions, if not legally punished, are viewed in the light of fools, and laughed at by the mass of the nation." Under such circumstances it would be vain to look for unbiassed arguments or discussions from the Spanish profession. We must therefore trust to the observations of unprejudiced spectators from our own or other countries.*

Previously to exhibiting a brief sketch of the Barcelona epidemic of 1821, our author prefixes some observations on the state of the weather, and the prevailing forms for the half year preceding the rise of that destructive malady. These we must pass over, but the following short extract presents a kind of recapitulation.

"The extraordinary changeable state of the weather, the prevalence of diseases uncommon at other seasons, the severity of catarrhal, rheumatic, pneumonic, and other febrile affections, the numerous miscarriages, the sudden deaths of persons apparently in health, the suffocation of individuals who were exposed to mephitical vapours in the necessaries of the city, the sudden and inexplicable creation of some classes of animals, and the unaccountable destruction of others, were circumstances which foreboded an impending evil, which neither the ingenuity of man, nor any power which he has within his reach, could avert." 27.

The medical faculty could not correctly ascertain the precise period when the common endemic bilious remittent fever ceased, and the real epidemic began—no inconsiderable proof, as our author properly remarks, of the analogy which subsists between the two diseases. The first cases which appeared in 1821 passed unnoticed, but as the disease became aggravated, and when it was discovered that a formidable malady reigned in the place, then some of the physicians and most of the people were unwilling to acknowledge that a disease of such malignity could have its origin among them. A tale of importation, therefore, was conjured up, and the awful visitation was ascribed to intercourse with the infected subjects of shipping, &c. A Neapolitan sloop of war, from cruising in the Mediterranean—a brig from the Havannah—a vessel from Cadiz—two vessels from Mahon, and several craft in the harbour, from various points of the compass—these were pitched upon as the pestiferous agents in the epidemic! We need not accompany our author in his refutation of these ridiculous stories, as no rational physician in this country will give any ear to them.

"The authorities, perceiving the slow and gradual progress of

---

* After the Jesuitical conduct of the Court of France towards Spain, we can put little confidence in the *official* reports respecting the contagious character of the Spanish fever which have been published in Paris.

the malady, and willing to make the public believe that it was wholly confined to the port, declared in the public papers, that they had it within their control, and consequently that it should be brought to obedience. The promise was bold, but not wise. Force of arms was of no avail; the disease started up in different parts of both towns, without a possibility of ascertaining from whence it came. The futility of guards became obvious; the troops were removed from the port, and the gates of the city were closed against the inhabitants of Barceloneta. After that was done, the malady spread with extraordinary rapidity in Barceloneta, and in that part of Barcelona which skirts the port: scarcely an individual escaped who was long exposed to the atmosphere of these localities; for there the morbific cause would seem to have been concentrated in a singular degree. From Barceloneta the disease marched in a westerly course through the centre of Barcelona, diminishing in activity as it proceeded outwards. It was finally lost before it reached the north-west extremity of the city; and, beyond a given limit, the epidemic character ceased, or only appeared by accident." 35.

Our author states, in the next few pages, the circumstances which lead to the rejection of the doctrine of importation. They are strong and numerous—and, as far as this fever is concerned at least, they are quite conclusive.

All the world has heard of the famous French commission sent by the Cabinet of the Tuilleries to the South of Spain—not to disprove contagion, it may be assumed, but to countenance the mask worn by the "Cordon Sanitaire." Messrs. Pariset, Bally, and Francois, in their report to the political chief of Catalonia, assert, 1st, that the fever was (of course) foreign or exotic of Spain, and imported into that country—2dly, that it was contagious, or capable of propogating itself afterwards, a quality that need hardly be questioned if it was capable of being carried across the Atlantic—3dly, that the disease was a perfect proteus, and therefore that it was impossible to lay down any plan of treatment. In short, they acknowledge that profuse *spontaneous* perspirations were the best kind of physic—and wisely conclude, that a good health police is infinitely preferable to the most excellent methods of treatment—"because in every country it is much better to have no sickness than good physicians." It is proper here to state that, in a private letter to a friend at Madrid, Dr. Pariset lets out the following curious piece of information:—"In fact, my beloved friend, *the malady is so in veterate and fatal, that there is no facility for the dissection of bodies, as one would desire, nor of remaining in the hospital the necessary time, in order to observe deliberately the disorder.*" What an acknowledgment from men who took upon themselves to solve such weighty questions!

"A ward was allotted for the French commissioners in the Seminario hospital, and adequate means of carrying into effect an effective system of treatment were afforded to them. They entered upon the task with eagerness, but not without using the precaution of covering themselves with oil-cloth dresses. This necessarily struck terror into the hearts of the sick, who naturally imagined, that where such means of preservation were employed by the faculty against the impressions of contagion, their own chances were almost desperate. The results of the practice of the French physicians were not successful; and the Spanish doctors now boast of one consolation, that, if they only cured one patient in ten, the French were still behind them; for there were, in fact, but very few recoveries in their wards.

"Under those circumstances, I cannot help expressing my astonishment at their effrontery, in making observations on the cure of a disease, which set their art at nought; and to which, in fact, they did not approach, except under circumstances which indicated that they were under impressions which obscured their judgment, and paralized their faculties; it is not therefore surprising that the French commissioners were unsuccessful practitioners." 68.

As for the manifesto of the Spanish Medical Junto, ycleped "the Medical Commission of Carthagena," it is a tissue of equivocations and absurdities bearing intrinsic evidence that the commission scarcely believed a word of what they published! These said commissioners only visited the hospital *once*, and at the time the report was issued, no official investigation had been instituted to ascertain the correct history of facts! In short, the whole train of transactions, Spanish and French, is too well calculated to shew how medical opinions have been subjugated by political despotism. Thank God, in England we are yet free to exercise our judgments without fear of ruling powers, and if we come to investigations with biases or prejudices, they are those of the individual, who would scorn to succumb to the dicta of political authorities.

The 3d chapter of the work before us contains "general observations on contagion," and we regret that we can give but a very limited view of these observations, as they are not very susceptible of analysis. Still we feel anxious to state some of the many important circumstances brought forward in this chapter.

It appears that the disease ravaged like a pestilence the cities of Barcelona and Tortosa, destroying thousands, without respect to age or sex;—while in the towns of Asco, Mequinenza, Malaga, Cadiz, Seville, and several other towns, its attacks were so limited as scarcely to deserve the name of epidemic. Now, our author argues thus:—

"Had the propagation of the malady depended upon specific contagion, or intercourse with the sick, the result, it is presumed, would have been pretty similar throughout the different towns in which the contagion appeared; for the most unlimited intercourse took place between the sick and healthy,"* 71.

As in each of the towns in which the minor epidemics prevailed, between two and seven hundred persons died of the yellow fever—and as some thousands, at least, must have been exposed to the contagion, if it did exist, it must be granted, that if the malady was contracted by intercourse, or near approach, the result ought to have been similar in all. The reverse was the case, apparently owing to some atmospheric or terrestrial difference.

"The disease was diffused rapidly in some; in others, where the cause existed in a minor degree, the friends and relations performed all the necessary offices for the sick, and attended carefully at their bed sides: the children slept with their parents, the wives, regardless of danger, slept in the very bed with their husbands; yet, the individuals who were thus exposed to the impressions of contagion, if contagion existed in the case, were not injured by the communication, even under circumstances apparently favourable to the diffusion of contamination." 73.

In the city of Barcelona, where the disease appeared, for the first time, in the year 1821, and where the physicians were, to a man, prejudiced in favour of contagion, a rigid scrutiny was nevertheless made, not only in private houses and public establishments, but also in the adjacent villages, country-houses, and lazarettos.

"And when they were convinced as to its true nature, from the strong proofs which daily offered to their observation, they were too candid, liberal, and enlightened, not to forego their former prejudices; and, with the candour and magnanimity of men of science and knowledge, they have, with few exceptions, avowed to the world, that the doctrine of contagion, in so far as relates to the disease called yellow fever, is founded upon fiction." 74.

During this destructive epidemic every kind of traffic went on extensively between Barcelona and the different provincial

---

* "The Spanish soldier of the present day is, probably, the most wretched object of the community. He is sometimes ill fed, ill paid, ill clothed, and not unfrequently destitute of the common means of support: he is always open to bribery. As admission is thus obtained to the bed-side of the sick, if it be desired, the preventive measure of the government is thus violated; and, as the lower orders of Spaniards evince a total indifference to the danger of visiting their sick friends, the most unlimited intercourse does or may take place. This is the fact: the orders to the sentinel are peremptory; they are disobeyed for a trifling sum of money."

towns—and yet all those towns and villages, with the exception of Tortosa, Asco, and Mequinenza, which are situated on the muddy banks of the River Ebro, at a distance of more than ninety miles from Barcelona, enjoyed absolute immunity from sickness.

"It is evident," says our author, "from what has been stated, that some other cause, besides contagion, must have been in action before the disease became epidemic; for, had the propagation depended upon specific contagion, all parts of the city of Barcelona would have been similarly affected: it is, on the contrary, known, that parts of it were healthy where no precautions were taken, and where no ostensible cause could be assigned for exemption; except that of distance from what appeared to be the focus of the cause, or by contingent protection from the force or free play of the pestilential atmosphere; which, having its origin in the eastern part, was obstructed and actually destroyed before it reached the north-west extremity of the city, where comparative good health prevailed. The almost absolute exemption of one part of the city from fever, under the circumstances described, proves that no contagion existed in the case, and that the epidemic cause was somewhat connected with the noxious vapours arising from the depositions of filth in the port and surrounding parts, for there the disease prevailed to an extraordinary degree." 89.

Many remarkable circumstances are next stated by our author, affording strong reasons for concluding that the epidemic in question possessed little, if any, contagious character. We shall only have room to quote one of these circumstantial facts.

The Casa Miserecordia, or House of Pity, is a building of considerable magnitude, situated in the immediate vicinity of the House of Charity, in the north-west extremity of Barcelona, in an apparently healthy situation. The building is low, but the apartments are spacious and well ventilated. It contained one hundred and fifty girls during the rage of the epidemic. The nuns who teach them are twenty-four in number. They maintain themselves by washing, ironing, and other similar modes of occupation. They employ women to traverse the city—from house to house, to procure needle-work, &c. These women went to all parts of the city; they communicated indiscriminately with the inhabitants; they were not affected by the disease; nor did the nuns or girls suffer by communicating with them." 93.

This, as Dr. O'Halloran observes, is a singular and a strong fact. For it certainly appears strange that a disease which was said to have been transported from the Havannah to Barcelona, and from Barcelona to other places, could not be introduced into the Miserecordia through such numerous channels of communication!

We cannot resist the desire to give the particulars of another, among the many instances of a nature similar to the foregoing.

The House of Correction, situated in the north-west extremity of the city, is a building well ventilated and with spacious and clean apartments. In this there were 100 females during the epidemic season. Nine of them were attacked with the reigning malady, and these were removed to the Seminario Hospital, where four of them died. The other five returned to the House of Correction, where they freely communicated with all the inmates, yet no more sickness ensued. This house was also visited by some of the female inhabitants of the city, who, through charity, brought eatables, &c. but no infection to the institution.

"It is unnecessary to multiply examples. Some hundreds may be produced to prove the noncontagious nature of the malady, more particularly in the country within the cordon, where the population was immense, owing to the emigrations from the city. I shall however, briefly observe, that the course of the malady from north-east to south-west, the different degrees of force attending its action in the course of its movements, its unusual destruction at what may be called the focus, during the whole of its continuance, the irregularity of its proceedings, jumping from point to point, and leaving intermediate places untouched; the diminished degrees of violence by which it was characterized as it proceeded towards the westward; its fatal and malignant nature in ill-ventilated places; the exemption of parts of the city from its influence, where no precautions were taken; the sickening of persons who observed the strictest seclusion; the sudden impression of contaminated air on persons recently from the country, without communication with the inhabitants of the city, the greater exemption of nurses and other attendants on the sick from the disease, than those who were simply exposed to the contaminated air of sickly houses; the almost absolute exemption of washers of bedding, clothes, &c., which had recently been used by the sick; the circumstance of the attendants in the hospitals and lazarettos having generally escaped the impression of the malady; the impossibility of diffusing the disease in the country, where no epidemic cause existed; and, finally, the deaths of some hundreds of persons who communicated with Barcelona, and who sickened in the neighbouring villages and country houses, without a solitary instance of its affecting the most assiduous of the attendants, however circumstanced, are ascertained facts, and convincing proofs of the non-contagious nature of the yellow fever." 101.

We must pass over several chapters that follow, on the medical topography and fever of Tortosa, Malaga, Puerto de Santa Maria, Xeres, Lebrixa, San Lucar de Baromada, and Cadiz, as we cannot do justice to our author's laborious investigations within the limits of this article. In the tenth chapter on Cadiz and Gibraltar, Dr. O'Halloran has made some sharp criticisms on Dr. Pym, and indeed has taxed him with certain erroneous statements which it will be indispensibly necessary for Dr. P. to notice, and explain if he can—more especially the statement in his (Dr. P.'s) book respecting the entire immunity which the inmates of the Dock-yard are said to have experienced in the year 1813, in consequence of cutting off all communication with the garrison and other parts of the rock. Dr. O'Halloran, in opposition to this, gives a list of eight deaths (from the fever we presume) which occurred in this secluded spot, out of a population of 170 souls, between the 18th September, and 23d December of the said year. A list of sixteen sick is also given, but the diseases are not specified. The charge is made against Dr. Pym with such gravity by our author, that the former cannot, we imagine, allow it to remain unnoticed.

In the "general remarks" which follow the 10th chapter, our author recapitulates most of the arguments, founded on the facts stated in the body of the work.

"From an impartial consideration of all the circumstances attending the epidemics of Spain, in the year 1821, the conclusion is, I think, fairly deducible, that the disease was not, and is not occasioned by imported contagion; and that its origin cannot be attributed to the germ of a former epidemic, resuming orignal activity from the operation of a peculiar state of the atmosphere, without which it would remain dormant perhaps for ever." 177.

On the contrary, he thinks that, would the Spaniards permit themselves to draw conclusions from an accurate knowledge of the general laws of the epidemics of the Mediterranean coast of Spain, and from the nature, construction, and situation of the towns, the conclusion would be irresistable:—

"*Viz.* that warm south-east breezes, unusual drought, excessive heat, want of ventilation in the houses, exhalations from swamps, lakes, &c., and, above all, the malignant effluvia which arise from the decayed remains of putrid animal and vegetable matters, with which the majority of the seaport towns of Spain abound, and which in calm and sultry weather, particularly in hot and oppressive nights, render the air impure, and offensive to the sense of smell,—are the causes which essentially influence or increase the activity of what may be called the seminium of epidemic yellow fever." 180.

The eleventh chapter is entitled "dissections," which are eleven in number. They all exhibit the ravages of the fever on various organs and parts, demonstrating the inflammatory nature of the complaint, and the necessity of antiphlogistic measures. The following extract will prove interesting to our readers.

" There are two points, particularly, in the foregoing dissections, which claim the attention of the reader. They unequivocally prove the highly inflammatory nature of the malady, and the cause of its unusual destruction. I believe that the internal state of the eye has rarely been the subject of post mortem researches ; and as I am of opinion, that its contents are more or less deranged by the effects of inflammation, when that organ is the seat of pain, accompanied with external redness : I also think, that the discovery is important, as proving to a demonstration the almost utter impossibility of removing the disease without the rigorous employment of the lancet, and other means of depletion.

" The state of the kidney, next to that of the eye, claims our attention. It is the general opinion, that this viscus is not liable to suffer from inflammation and its consequences in the yellow fever, even where suppression of urine is a fatal and prominent symptom for days. I have seen more than two hundred dissections, but I do not recollect having seen the kidney examined minutely, a single incision through the pelvis satisfied the examiner, who pronounced it healthy. In all the post mortem examinations which took place previous to my arrival at Barcelona, if I can credit the report of persons who assisted at them, little attention was paid to the kidney,— a single cut and no more. In my four first dissections I was equally careless with regard to its state, having been informed, that in no one instance did it exhibit marks of disease, although suppression of urine was a prominent symptom in almost all the fatal cases. In the case of Maria Gouch, I accidentally discovered abscesses : the existence of abscesses in the papillæ of the kidneys led to the examination of the ureters, which also contained pus. I cannot say that abscesses of the kidneys are common in the yellow fever of Andalusia, having only performed a few dissections there; but, as the symptoms of the disease in Barcelona (where I am disposed to think, from what I witnessed, that abscesses of the kidneys were common) bore considerable analogy to the inflammatory form of the Andalusian fever, I am inclined to believe that the same appearances exist, and will be found on dissection ; and as it is a most important point, as connected with the treatment, to ascertain whether or not the suppression of urine arises from inflammation of the kidneys and its consequences, I must take the liberty of calling the particular attention of anatomists to this important subject. Every atom of the viscus ought to be examined ; the abscesses are extremely small, and difficultly discovered. The liver in most cases exhibited unequivocal marks of recent disease, more so than I before witnessed. The post mortem examination of subjects, who die of the yellow fever, should take place as soon as possible; for the different organs, viscera, &c., more particularly those that have recently suffered from diseased action, undergo changes in a few hours, so as to become brown, black, and apparently gangrened, while the appearances of other parts, that were comparatively healthy, are also changed after a given time. I am disposed to think, that if the body be not examined in five hours after death, little information can be obtained of the actual state ; and that this, in a great measure, accounts for the extensive gangrenous appearances which anatomists have described, and which, in my opinion, rarely take place during life as the effect of disease; but as the vitality of parts, upon which a prominent morbid action exercises its injurious influence, is more or less in a state of debility from the effects of inflammation, when dissolution takes place, they more readily undergo changes and assume gangrenous appearances than healthy parts. This may be proved by exposing the abdominal contents as soon as possible after death. Then the changes are evident; for the parts, which have suffered from recent disease, change colour and become black in a very short time, whilst those, that have sustained no injury, retain the natural colour for a much longer period." 203.

In concluding this article, we acknowledge that we have not been able to do that justice to Dr. O'Halloran's publication, which its own merits and the zeal and talents of its author deserved. We hope, however, that we have done enough to excite all those whom it may concern to consult with care the original work. We have no doubt that the able and worthy author will not be overlooked by the commander in chief or the heads of his own department.

